# Commercial PRRS Modified-Live Virus Vaccines

**DOI:** 10.3390/vaccines9020185

**Published:** 2021-02-22

**Authors:** Chanhee Chae

**Affiliations:** Department of Veterinary Pathology, College of Veterinary Medicine, Seoul National University, 1 Gwanak-ro, Gwanak-gu, Seoul 08826, Korea; swine@snu.ac.kr

**Keywords:** modified-live virus vaccine, porcine reproductive, respiratory syndrome virus

## Abstract

Porcine reproductive and respiratory syndrome (PRRS) virus (PRRSV) presents one of the challenging viral pathogens in the global pork industry. PRRS is characterized by two distinct clinical presentations; reproductive failure in breeding animals (gilts, sows, and boars), and respiratory disease in growing pigs. PRRSV is further divided into two species: PRRSV-1 (formerly known as the European genotype 1) and PRRSV-2 (formerly known as the North American genotype 2). A PRRSV-2 modified-live virus (MLV) vaccine was first introduced in North America in 1994, and, six years later, a PRRSV-1 MLV vaccine was also introduced in Europe. Since then, MLV vaccination is the principal strategy used to control PRRSV infection. Despite the fact that MLV vaccines have shown some efficacy, they were problematic as the efficacy of vaccine was often unpredictable and depended highly on the field virus. This paper focused on the efficacy of commercially available MLV vaccines at a global level based on respiratory disease in growing pigs, and maternal and paternal reproductive failure in breeding animals.

## 1. Introduction

Porcine reproductive and respiratory syndrome (PRRS) has a long history from its first discovery and description in the United States in 1987 as a “mystery swine disease” [1]. Later, Europe named it in 1990 as “blue ear disease” [2]. Once the virus was isolated for the first time in the Netherlands in 1991, the causative agent was named “Lelystad virus” [3]. Following the isolation of Lelystad virus in Europe, a virus was also isolated in the US with resembling clinical field signs and named VR-2332 [4]. Additional names followed such as pig plaque 89, swine infertility and respiratory syndrome (SIRS), disease 89, swine reproductive and respiratory syndrome (SRRS), and porcine epidemic abortion and respiratory syndrome (PEARS). “Porcine Reproductive and Respiratory Syndrome (PRRS)” and its virus “Porcine Reproductive and Respiratory Syndrome Virus (PRRSV)” finally received its official name at the 1992 First International Symposium on SIR/PRRS held at St. Paul, Minnesota (USA). PRRSV has been reclassified to the *Arteriviridae* family (genus *Porartevirus*) in the order Nidovirales together with the following families: *Coronaviridae*, *Roniviridae*, and *Mesoniviridae* [5,6]. Due to its high degree of genetic diversity, PRRSV was further divided into two species, PRRSV-1 (formerly known as the European genotype 1) and PRRSV-2 (formerly known as the North American genotype 2) [5,7].

PRRS has had an enormous economic impact on the pig industry and is considered to be one of the most challenging diseases to manage worldwide due to the unpredictable efficacy of available modified-live virus (MLV) vaccines. PRRS is characterized by two distinct clinical presentations; reproductive failure in breeding animals, and respiratory disorders which predispose growing pigs to secondary infections associated with porcine respiratory disease complex. Reproductive failure in gilts and sows was characterized by abortion and delivery of stillborn, near-term fetuses, or premature and weak piglets. Reproductive failure in boars was characterized by clinical manifestations which included anorexia, lethargy, and a loss of libido [8].

At the present, “perfect” PRRS vaccine is not yet commercially available. All vaccines are based on live (MLV) or killed virus and have advantages and disadvantages [9,10,11,12,13]. The major advantage of killed vaccines is safe but they confer limited efficacy against homologous and heterologous virus, in particular, in naïve animals [11,12]. On the other hands, the major disadvantage of MLV vaccines is safe but they confer complete protection against homologous virus and partial protection against heterologous virus [9,12,13]. For these reasons, MLV vaccines are considered to be more efficacious than killed vaccines [11]. Consequently, MLV vaccines are the predominating vaccines in the field nowadays. It is also worthy of note that the effectiveness of MLV vaccines in the field depends not only on the biological properties of the vaccine itself, but also on vaccination strategy how the vaccine is applied and what other biosecurity measures are in place [14].

The first PRRSV-2 MLV (MLV2) vaccine was introduced in North America in 1994. Six years later, a PRRSV-1 MLV (MLV1) vaccine was also introduced into Europe. Both types of MLV vaccine have since become the principal means used to control PRRSV infection. Different types of MLV vaccines are needed on different continents due to both the distribution of the PRRSV species and the disease severity (e.g., reproductive failure, respiratory disease, or both). Europe, for example, uses the same vaccine for both the Eastern and Western geographical regions, but the disease profile is diverse between two European regions. PRRSV-1 subtype 1 infection in Western Europe is mainly associated with reproductive failure in sows [15]; therefore, MLV1 is mainly used to control reproductive failure. Meanwhile, the highly virulent PRRSV-1 subtype 3 is linked with both respiratory and reproductive problems in Eastern Europe, resulting in large economic losses. The same MLV1 is used to control both reproductive failure and respiratory disease [16]. The occurrences of PRRSV-1 outbreaks in North America are relatively rare, so MLV-2 is mainly used to control reproductive failure and respiratory disease. The PRRS situation of Asia is unique to those of Europe and North America. PRRSV-2 is the more predominant virus in Asia, although PRRSV-1 is simultaneously prevalent, with both viruses causing reproductive failure in sows and respiratory disease in growing pigs. MLV1 and MLV2 are both widely used, consequently, to control reproductive failure and respiratory disease in Asian countries.

Many review articles have already been published on the protective immune mechanisms of commercial MLV vaccines [9,10,12,13] but there has been less discussion of the overall efficacy of commercial MLV vaccines against various PRRSV strains from different countries. Therefore, this review focuses on the efficacy of commercially available MLV vaccines at the global level and evaluates efficacy in terms of maternal (sow) and paternal (boar) reproductive failure, and respiratory disease in growing pigs.

## 2. Respiratory Diseases

### 2.1. Criteria of Vaccine Efficacy for Respiratory Disease

Virus replication and respiratory disease were considered for the assessment of the vaccine efficacy. Following the respiratory route of infection, PRRSV replicates in the respiratory tract and causes viremia and dissemination throughout the body of the animal. PRRSV viremia therefore plays a key role in the development of respiratory diseases [17,18]. A close correlation was observed between the amount of viral load in the blood and the severity of lung lesions [17,18]. Reduction of viremia is a useful indicator for assessing the efficacy of a MLV vaccine as it is linked to both viral spread reduction and a reduction in lung lesions [17,19]. Although the reduction of PRRSV viremia is a critical parameter for the evaluation of PRRSV vaccines, the mechanisms of the viral clearance are still poorly understood. PRRSV viremia is often resolved even before neutralizing antibodies (NA) are detected in infected pigs [18,20,21,22] and vaccinated pigs [23,24,25,26]. Despite the low NA levels in response to PRRSV vaccination [23,24,27], pigs that received a MLV vaccine followed by a PRRSV challenge still efficiently cleared PRRSV in the blood. Presumably, this was at least partially dependent on cell-mediated immunity, especially the host interferon (IFN)-γ response. IFN-γ is known to inhibit the replication of PRRSV in macrophages and is able to trigger specific T cell proliferation and cytotoxic immunity activation [28,29,30,31]. Measurement of IFN-γ secreting cells (IFN-γ-SC) is a frequently used tool for evaluating the recall IFN-γ responses after vaccination or infection. Numerous vaccinate-challenge studies found a significant negative correlation between the IFN-γ response and blood viral load, indicating induction of IFN-γ responses by MLV vaccine may result in the reduction of viremia [23,25,26,32,33,34,35]. Cell-mediated immune response is therefore an important component in the clearance of PRRSV viremia.

The efficacy of MLV vaccines can be evaluated based on (i) clinical parameters such as clinical signs, (ii) virological parameters such as amount of viral load in the blood, (iii) immunological parameters such as IFN-γ response, and (iv) pathological parameters such as lung lesions and amount of viral load in lungs under experimental and field conditions.

### 2.2. PRRSV-1 MLV Vaccines

PRRSV-1 subtype 1 strains circulating in Western Europe, Asia, and North American are predominantly much lower pathogenically than the PRRSV-1 subtype 3 strains found in Eastern Europe. “Lena”, one of these highly virulent PRRSV-1 subtype 3 strains, was involved in the 2006 Belarus outbreak of severe reproductive and respiratory disorders associated with high mortality [16]. A strain of a PRRSV-1 subtype 2 (prototype “Bor”) has also been proven to be more virulent than PRRSV-1 subtype 1 [16]. The Western European PRRSV-1 situation has rapidly evolved in the last ten years. During this time, both Italy and Belgium reported the emergence of a highly pathogenic PRRSV-1 subtype 1 strain [35,36]. The reports altered the previous pattern of a geographical demarcation between Western areas with low pathogenic PRRSV-1 subtype 1 (Lelystad-like) strains and Eastern European areas with highly pathogenic PRRSV-1 subtype 3 (Lena-like) strains.

**Figure 1:**
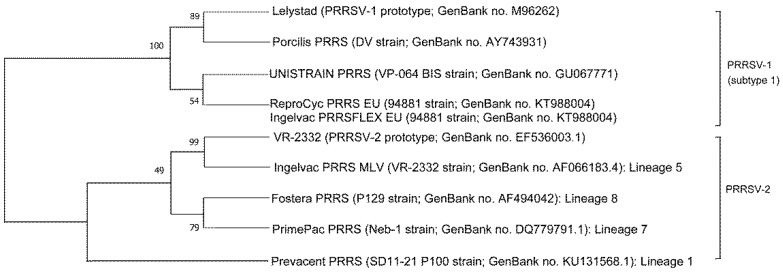
Phylogenetic analysis. Open reading frame 5 genome from the vaccine viruses with prototype of Porcine Reproductive and Respiratory Syndrome Virus (PRRSV)-1 (Lelystad) and PRRSV-2 (VR-2332). An unrooted neighbor-joining tree was constructed from aligned nucleotide sequences.

Four MLV1 vaccines based on subtype 1 are commercially available and currently used in European and Asian countries (Table 1) (Figure 1). Numerous articles have been reported in efficacy of MLV1 vaccines against respiratory disease (Table 2). Vaccination of pigs with a MLV1 vaccine resulted in reduction of viremia levels and reduction of lung lesion severity following experimental infection with a heterologous Lelystad-like (subtype 1) strain [37,38,39,40]. In addition, a MLV vaccine provided partial protection against Italian highly pathogenic PRRSV-1 subtype 1 strain based on clinical, virological, and pathological analysis [41]. All four MLV1 vaccines tested provided virtually the same clinical partial protection against infection from the highly pathogenic PRRSV-1 subtype 3 Lena strain by reducing the fever period duration in all studies [32,33,34]. While evaluating virological parameters, a significant reduction in viremia with two MLV1 vaccines was shown [32,34] while a significant decrease in viremia was not observed in the remaining two MLV1 vaccines [33,34]. Although a direct comparison of the efficacy of vaccines tested in different studies is difficult, it appears that MLV1 vaccines based on PRRSV-1 subtype 1 provided better protection against the same subtype 1 challenge than against a subtype 3 challenge.

Minimal peer-reviewed information exists in regard to the MLV1 vaccine efficacy against PRRSV-2 because of the minor pathogenic role of PRRSV-2 in Europe. Field wild type PRRSV-2 strains, (unrelated to MLV2 vaccine virus), have been reported in Hungary, Germany, and Greece [42,43,44]. In general, MLV1 vaccines provide limited protection against PRRSV-2. Nevertheless, the efficacy of MLV1 vaccines could be different and is dependent on the challenge PRRSV-2 strain used [37,45]. Vaccination of pigs with MLV1 did not reduce the level of viremia and lung lesions after challenge with PRRSV-2 [37,46,47] while the same MV1 vaccine did reduce the levels of viremia and lung lesions post-challenge with a different PRRSV-2 strain [45,47]. Good efficacy of a MLV1 vaccine against one PRRSV-2 strain does not guarantee the same level of efficacy against another PRRSV-2 strain. These results suggest that MLV vaccine efficacy may depend on the challenge virus. The efficacy of MLV1 vaccines against various PRRSV-2 challenge strains is therefore unpredictable and additional testing is needed.

### 2.3. PRRSV-2 MLV Vaccines

Currently, three of the four evaluated MLV2 vaccines are used worldwide while the fourth MLV2 vaccine, Prevacent PRRS, has only recently been introduced into the United States (Table 1). The first MLV2 vaccine virus that was introduced to the market belongs to lineage 5, whereas the sequential three other MLV2 vaccines belong to lineages 8, 7, and 1, respectively (Figure 1). Numerous articles have been reported in efficacy of MLV2 vaccines against respiratory disease (Table 2). In general, pigs vaccinated with MLV2 exhibited some efficacy after a heterologous PRRSV-2 challenge [23,48]. The MLV2 vaccine first introduced into the market provided good protection against a Korean heterologous PRRSV-2 strain [49]. Interestingly, after 20 years of use in Korea, the same MLV2 vaccine is still proven to be efficacious against recently isolated heterologous PRRSV-2 strains [50]. These results provide swine practitioners and producers with clinically significant information as the rapid evolution of PRRSV is an important driving force for the emergence of new strains capable of vaccine resistance [51]. The second MLV2 vaccine introduced into the market has been shown to reduce viremia levels and nasal shedding as well as reduce the severity of PRRSV-induced lesions following a challenge with a variety of PRRSV-2 strains from Asia and North America [23,26,48,52].

Highly virulent PRRSV has started to emerge as a worldwide threat, in particular, a highly pathogenic (HP)-PRRSV strain based on PRRSV-2, originating from China [53,54,55]. HP-PRRSV-2, known as pig high fever disease, was first reported in 2006 in China and has spread rapidly to neighboring southeastern Asian countries [4,53,54,55,56,57]. Infections with HP-PRRSV-2 are characterized by high fever (40–42 °C) and high mortality (20–70%) of young and adult pigs [53,54,55]. Two MLV2 vaccines have proven efficacious in protecting growing pigs against a HP-PRRSV-2 challenge [58,59,60,61,62]. Among two vaccines, one MLV2 vaccine (Fostera PRRS) provided slightly better protection (body temperature, levels of viremia, and number of IFN-γ-SC) against HP-PRRSV-2 of the same genomic lineage when compared to the other MLV2 vaccine (Ingelvac PRRS MLV) of a different genomic lineage [60]. These differences between two MLV2 vaccines may suggest that the former MLV2 vaccine virus and challenge HP-PRRSV-2 that are closely related genetically may be also closely related antigenically. MLV1 vaccination, by contrast, offered a limited to partial protection against HP-PRRSV-2 challenge [47,63]. These results indicate that MLV2 vaccines are more efficacious in the control of HP-PRRSV-2 infection in pigs when compared to MLV1 vaccines.

MLV2 vaccines afford significant protection in a challenge with a PRRSV-1 strain [25,49]. MLV2 vaccine has been shown to reduce viremia and lung lesion levels post-challenge with Korean and European heterologous PRRSV-1 subtype 1 (Lelystad-like) strains [49,64] but was not able to reduce the levels of viremia against European heterologous PRRSV-1 subtype 1 (Lelystad-like) strains [46,65]. These results suggest that cross-protection of MLV2 vaccines against PRRSV-1 is inconsistent. In addition, MLV2 vaccine reported partial protection (a decrease in clinical and virological parameters) against a highly pathogenic PRRSV-1 subtype 3 Lena strain [34].

### 2.4. PRRS-1 and PRRSV-2 MLV Vaccine against Co-Challenge

Both PRRSV-1 and PRRSV-2 are concurrently circulating in several Asian (Korea, China, Vietnam, and Thailand) pig farms, causing respiratory diseases in growing pigs [12,13,63,66]. Co-infection with both species of PRRSV is increasing and prevalent (14.6%; 73 out of 500 cases) in diagnostic respiratory cases [67]. Therefore, control of both species by one MLV vaccine is the clinical ideal as producers prefer to reduce the number of vaccinations in growing pigs.

PRRSV-1 and PRRSV-2 strains differ in their ability to replicate in the pigs dually infected with both PRRSV-1 and PRRSV-2 [67]. Consequently, PRRSV-1 is unable to exacerbate interstitial pneumonia in dually infected pigs. Pigs dually infected with PRRSV-1 and PRRSV-2 developed similar clinical disease and lesions as pigs that were infected with PRRSV-2 alone [67]. For this reason, an emphasis should be placed on a MLV vaccine that controls PRRSV-2 rather than PRRSV-1 in dually infected pigs. MLV2 vaccination was efficacious in protecting growing pigs from respiratory disease after a dual challenge when compared with MLV1 [68]. The MLV2 vaccine induced a higher number of PRRSV-1 and PRRSV-2 specific IFN-γ-SC compared to the MLV1 vaccine after a dual challenge. These differences may explain why MLV2 vaccination is more effective against a dual challenge when compared to MLV1 vaccination [68]. These findings are consistent in field studies, where the MLV2 vaccine is also effective against respiratory disease in PRRSV-1 and PRRSV-2 concurrent endemically infected farms [69,70,71]. This dual challenge study contradicted another study, where a significant difference between MLV1 and MLV2 against dual PRRSV-1 and PRRSV-2 challenge did not occur [72]. The discrepancy between two dual challenge studies may be due to the different challenge virus used per study.

### 2.5. Co-Vaccination of PRRS-1 and PRRSV-2 MLV Vaccine

PRRSV-1 and PRRSV-2 are the predominant species in the European and North American continents, respectively, while the situation for most Asian countries including Korea is more complicated as both PRRSV-1 and PRRSV-2 are prevalent and have been shown to cause disease [67]. Theoretically, one possible way to control co-infection of pigs with two species may be the concurrent vaccination of pigs with both MLV1 and MLV2 vaccines as a combined vaccine containing PRRSV-1 and PRRSV-2 is not yet commercially available. One study reported that co-vaccination of pigs at four weeks of age provided only partial protection against respiratory disease caused by a dual challenge with PRRSV-1 and PRRSV-2 [73]. Another study contradicted these finding as concurrent vaccination could only provide protection against respiratory disease caused by PRRSV-1 in four-week-old pigs with a dual challenge [74]. In the latter study [74], co-vaccination of pigs with MLV1 and MLV2 vaccines significantly hampered the efficacy of the MLV2 vaccine but not the MLV1 vaccine. The two compared studies did use different commercial MLV1 and MLV2 vaccines. These genetic differences between vaccine and challenge virus could play a role on the efficacy of the vaccine and have caused the discrepancy between the two studies. Further studies are needed to elucidate the cause of discrepancy.

## 3. Maternal Reproductive Failure

### 3.1. Pathogenesis of Maternal Reproductive Failure

Gestation time plays a crucial role in pregnant sow infection. In early gestation, PRRSV has be known to cause embryonic death in early gestation [75,76]. In mid-gestation, PRRSV has a minimal impact on reproductive failure as the virus does not readily cross the placenta [77,78], but this changes during late gestation which results in abortions, early farrowing, fetal death, or the birth of weak, congenitally infected piglets that contributes to an increase in pre-weaning mortality [78,79,80,81].

Both PRRSV-1 and PRRSV-2 have equal pathogenicity during late gestation [82]. Although PRRSV does not cross the placenta in early and mid-gestation, this capability occurs in late gestation [77]. When PRRSV crosses the placenta during this late gestational period, the litter is exposed to the virus from the infected sow by placental infection [83,84]. Although the degree of maternal viremia does not directly cause abortions [83], it plays an important role in the placental crossing of PRRSV along with its replication in the endometrium [85]. It is likely that placental PRRSV infection induces pathological lesions in the maternal–fetal interface [86], resulting in placental degradation and a deterioration of placental function [87]. This enables the spread of PRRSV to the fetal tissue and results in the fetuses becoming infected [83,84]. Once the virus enters the fetus it quickly spreads to multiple fetal tissues [83,88]. Of these fetal tissues, the thymus is the primary site of viral replication in the fetus of a PRRSV-infected gilt [82,89]. A high viral load in certain tissues, especially in the fetal thymus, increases the chance of fetal death [83,88].

### 3.2. Criteria of Vaccine Efficacy for Maternal Reproductive Failure

The evaluation and comparison of MLV vaccines in sows is important as most Asian pig farmers vaccinate sows more readily than they do growing pigs. PRRV primarily affects the late gestation stage as it is able to cross the placenta in this stage only [77,78,90] Reproductive symptoms that appear as a result of PRRSV infection such as premature farrowing occur later in gestation [8]. It is therefore reasonable and useful to determine the parameters for the evaluation of reproductive vaccine efficacy based on the pathogenesis of late-gestational PRRSV infection.

Improvement of reproductive performance is the most critical parameter in evaluating a MLV vaccine for the control of reproductive failure. Additional protective parameters are based on the pathogenesis of late gestation PRRSV infection. Although the virus is spread from fetus to fetus; a method that is independent of maternal levels of PRRSV viremia [83,91], transplacental infection correlates with levels of maternal viremia [83,84]. A significant reduction on PRRSV maternal viremia during early infection should therefore correlate better with protection and be considered as a protective parameter in the evaluation of reproductive efficacy of PRRSV vaccines. How the protective immune response results in the reduction of maternal viremia is not yet clear. Neutralizing antibodies play an important role in the protection against experimental challenge, where protection depends on the levels of neutralizing antibodies [92,93]. The protective role of NA is, however, limited in PRRSV vaccination [27]. Despite the low levels of NA elicited in response to PRRSV vaccination, vaccinated sows are still able to efficiently clear PRRSV viremia [94,95,96,97,98,99,100,101,102]. It is presumed that this was at least partially dependent on cell-mediated immunity, especially the host IFN-γ response, as IFN-γ is known to inhibit PRRSV replication [28,29]. Moreover, the reduction of PRRSV viremia coincides with the appearance of PRRSV specific IFN-γ-SC [94,96,103]. Therefore, PRRSV specific IFN-γ-SC may play a critical role in the clearance of PRRSV viremia. Detection of PRRSV in the fetal thymus is another clinically significant criterion in evaluating the efficacy of a PRRSV vaccine as the presence of PRRSV, (particularly at high levels in the thymus), contributes to fetal death [83]. The reproductive efficacy of a PRRSV vaccine is evaluated on the basis of the following three parameters: (i) the improvement of reproductive performance, (ii) the reduction of maternal viremia and the induction of IFN-γ-SC, and (iii) the reduction of viral load in fetal thymus.

### 3.3. PRRSV-1 MLV Vaccines

Numerous articles have been reported in efficacy of MLV1 against reproductive failure (Table 3). Vaccination is a key component in the reduction of the severity and frequency of reproductive complications related to PRRSV. Vaccination of gilts and sows with a MLV1 vaccine is beneficial for several reasons including improved farrowing and weaning rates and to decrease the number of premature farrowing instances [96,99,100]. MLV1 vaccines conferred partial to improved protection and improved the reproductive performance in gilts and sows against a heterologous PRRSV-1 challenge from Korea and Europe [96,99,100,101,102]. A field study confirmed that the same MLV1 vaccine also provided a beneficial effect on swine health and fertility in PRRSV-1 endemically infected farms [104,105].

Vaccination with a MLV1 vaccine is more efficacious against PRRSV-1 than PRRSV-2 [96]. Vaccinating gilts with a MLV1 vaccine can provide good protection against a heterologous PRRSV-1 challenge [96]. Reproductive performance was improved by MLV1 vaccination against PRRSV-1 [96]. Vaccinations of gilts with the PRRSV-1 MLV vaccine lead to a significant reduction of PRRSV-1 viremia which coincided with the appearance of PRRSV-1 specific IFN-γ-SC. Although the role of IFN-γ-SC is not yet known in its entirety, IFN-γ-SC is responsible for the reduction of viremia [96]. MLV1 vaccination confers limited cross-protection against PRRSV2 [96]. Vaccination of gilts with the MLV1 vaccine could not lead to a significant reduction of PRRSV-2 viremia. Failure to reduce PRRSV-2 viremia may be one of the reasons why the MLV1 vaccine provides only a limited protection against PRRSV-2 [96]. Interestingly, the same MLV1 vaccine was able to provide partial cross-protection in growing pigs against respiratory disease from a heterologous PRRSV-2 challenge (different strain) [47,49]. These results suggest that protection provided by a MLV1 vaccine against PRRSV-2 in respiratory symptoms does not necessarily mean protection against the same PRRSV-2 in reproductive disorders.

### 3.4. PRRSV-2 MLV Vaccines

Numerous articles have been reported in efficacy of MLV2 against reproductive failure (Table 3). MLV2 vaccines confer partial protection against a heterologous PRRSV-2 challenge from Asia and North America [94,95,98]. The vaccination of pregnant sows with MLV2 vaccine improves reproductive performance, reduces maternal viremia, and induces IFN-γ-SC response, while reducing the level of PRRSV-2 in the thymus of fetal pigs against a heterologous PRRSV-2 challenge [94,95]. This was consistent with a previous field study, where the same MLV2 vaccine was also efficacious against reproductive failure in PRRSV-2 endemically infected farms [106].

Cross-protection was measured, as vaccination of gilts with a MLV2 vaccine improved reproductive performance parameters such as number of live-born and number of weaned piglets post-PRRSV-1 challenge [103]. Cross-protection with MLV2 vaccines against PRRSV-1 is inconsistent. In the comparative study for two vaccines, one MLV2 vaccine provided good cross-protection, another MLV2 vaccine limited cross-protection against a heterologous challenge with the same PRRSV-1 strain [95,103]. These results suggest that the degree of protection by one MLV2 vaccine is not a valid indicator of the protection degree of another MLV2 vaccine. Moreover, MLV2 vaccine provide limited cross-protection in reproductive failure but good cross-protection in respiratory disease against a heterologous challenge with the same PRRSV-1 strain [49,95]. These results also suggest that good efficacy of MLV2 vaccine in respiratory disease does not guarantee the same level efficacy in reproductive failure against the same PRRSV-1 strain.

### 3.5. PRRS-1 and PRRSV-2 MLV Vaccine against Co-Challenge

As co-infection of PRRSV-1 and PRRSV-2 is continuously becoming more prevalent in Asian pig farms [97,107,108], swine producers and practitioners are trying to select proper MLV vaccines that can cross-protect against both PRRSV species. A bivalent MLV vaccine containing both PRRSV-1 and PRRSV-2 is currently commercially unavailable. To achieve the largest benefit from the commercial vaccines that are available, gilt and sow vaccination with a MLV2 vaccine is more effective against reproductive failure as evaluated by a dual heterologous PRRSV-1 and PRRSV-2 challenge during late-term pregnancy when compared with a MLV1 vaccine [107].

### 3.6. Co-Vaccination of PRRS-1MLV and PRRSV-2 MLV Vaccine

MLV2 vaccines can cross-protect against PRRSV-1 in terms of reproductive failure [103]. Nevertheless, MLV1 vaccines always provide better protection against a PRRSV-1 challenge [96]. Unfortunately, MLV1 vaccines did not provide protection against reproductive failure caused by PRRSV-2 in late-term pregnancy gilts whereas the MLV2 vaccine was very effective [94,96]. When all of these studies are placed together, the results suggest that MLV vaccines protect better against a challenge with the same species as the vaccine strain. Therefore, concurrent vaccination with MVL1 and MLV2 vaccines may provide complete protection against reproductive failure from PRRSV-1 and PRRSV-2 in sows and gilts [97]. This co-vaccination in sows contrasted the results from another study where co-vaccination only provided protection against respiratory disease caused by PRRSV-1 in 4-week-old pigs that received a dual challenge [74]. There is one possibility for the discrepant results. The different outcomes could be due to age-related immune responses. It appears that co-vaccination is more efficacious in adult pigs (i.e., gilts and boar) as their immune systems are mature enough to have a simultaneous immune response to each vaccine. A co-vaccination in younger weaning pigs (less than five weeks old) may cause physiological changes that can be detrimental to the cellular immune response of the animal [109]. Similarly, the age of the host can influence the dynamics of PRRSV infection [110]. Further studies are needed to elucidate these hypotheses.

## 4. Paternal Reproductive Failure

### 4.1. Pathogenesis of Paternal Reproductive Failure

PRRSV-1 and PRRSV-2 cause reproductive failure in boars with similar virulence [111]. Both PRRSV-1 and PRRSV-2 are able to replicate and induce apoptosis in the epithelium of the seminiferous tubules, producing alterations in the reproductive tract [112,113]. Reproductive manifestations in infected boars are considered as a loss of libido, and alterations in semen quality. Alterations in semen quality are further defined as a decrease in sperm motility, an increase in morphological anomalies, and spermatozoa with abnormal acrosome [114]. Infected boars have been found to shed PRRSV in semen for as short as four and as long as 92 days following experimental infection [115,116]. PRRSV in semen is transmissible to sows [117,118,119,120].

### 4.2. Criteria of Vaccine Efficacy for Paternal Reproductive Failure

Seminal shedding of PRRSV plays a major role in the transmissibility of the virus into sows [117,118,119,120]. The transmission of PRRSV via semen to offspring by artificial insemination has been reported [121]. The most important efficacy parameter of MLV vaccine evaluation is the ability to reduce the amount of PRRSV seminal shedding, as seminal transmissibility of PRRSV is dependent upon the viral load [122]. Reduction of viral shedding in semen may be related to the cellular immune responses induced by the MLV vaccine. Reduction of viral seminal shedding coincides with the appearance of PRRSV-specific IFN-γ-SC [123]. Therefore, induction of PRRSV-specific IFN-γ-SC by the MLV vaccine is one of the main factors leading to the reduction of seminal viral shedding in infected boars.

Vaccinated boars shed the vaccine viruses from two MLV vaccines for the first 21 days post-vaccination [123]. It should be noted that boar vaccination is acceptable in positive herds, but never an option in negative herds and will not securely guarantee PRRSV-free semen. Therefore, PRRSV vaccination in boars is an alternative method to help reduce shedding of PRRSV in semen when negative boars are unexpectedly infected with PRRSV in PRRSV-positive herds.

### 4.3. PRRSV-1 MLV Vaccines

Vaccination of boars with MVL1 vaccines significantly reduced the PRRSV-1 load amount in both blood and semen after PRRSV-1 challenge but rarely reduces the amount of the PRRSV-2 load in either the blood or semen following a PRRSV-2 challenge [124]. The frequencies of PRRSV-1 specific IFN-γ-SC induced by MLV1 vaccination are relatively high compared to the PRRSV-2 specific IFN-γ-SC induced by the same vaccine. This may explain why MLV1 vaccination is more effective in reducing seminal shedding of PRRSV-1 when compared to PRRSV-2 in vaccinated-challenged boars. These results indicate that MLV vaccines are more effective against the same PRRSV species rather than different species in terms of seminal shedding of PRRSV in infected boars.

### 4.4. PRRSV-2 MLV Vaccines

The vaccination of boars with MLV2 vaccine decreased subsequent shedding of the PRRSV-2 post-challenge but was unable to decrease the shedding of the PRRSV-1 in the semen following challenge [125]. These observations contradict a Danish study [126] in which PRRSV-1 shedding was significantly reduced after a heterologous challenge in boars that were immunized with the same MLV2 vaccine. There is no clear explanation for this discrepancy, but the antigenic variation between the Korean and Danish PRRSV-1 strains may play a role. The ORF5 and ORF7 nucleotide sequences in the Korean PRRRSV-1 are 88% and 91% identical, respectively, to the Danish PRRSV-1 isolate [125,126]. This genetic difference may indicate that the two PRRSV-1s are antigenically different.

### 4.5. Co-Vaccination of PRRS-1 and PRRSV-2 MLV Vaccine

Concurrent vaccination of boars with MLV1 and MLV2 vaccines significantly reduced the amount of both PRRSV species loads in both the blood and semen against both a singular and dual PRRSV challenge [123]. These results are similar to those of previous studies in sows [97].

## 5. Conclusions

The most defining characteristic of MLV vaccination is unpredictable efficacy. First, many researchers and practitioners predict the efficacy of a MLV vaccine based on the genetic homology between the vaccine and field virus. This has proven to be an inadequate practice; however, as the ability of a vaccine to protect against a certain field virus is not linked to the level of genetic homology. [13,57,127,128]. Secondly, good efficacy of a MLV vaccine against one PRRSV strain does not guarantee the same level of efficacy against a different PRRSV strain within the same country [35,45]. Third, one MLV vaccine may provide good cross-protection while the other MLV vaccine provides limited cross-protection against the same PRRSV strain [95,103]. These results suggest that the degree of protection by one MLV vaccine is not a valid indicator of the protection degree of another MLV vaccine. Fourth, one MLV vaccine may confer limited protection in reproductive failure but good protection in respiratory disease against the same PRRSV strain [49,95]. These results also suggest that good efficacy of a MLV vaccine in respiratory disease does not guarantee the same level of efficacy in reproductive failure against the same PRRSV-1 strain.

MLV vaccines are widely used to decrease PRRS-associated losses. The major advantage of MLV vaccines is their ability to elicit a protective immune response which mimics that of PRRSV infection. Ideally, MLV vaccine must have heterologous protective efficacy at least equivalent to homologous protection. However, MLV vaccines are unable to elicit a strong protection for animals against genetically and antigenically different field strains. Additional researches require to overcome the limitations of current MLV vaccines. On the other hands, despite the fact that only a limited number of cases have reported this safety concern with one particular vaccine to date [129,130], the largest concern with any MLV vaccine is their ability to revert to virulence [10,12].

Continuous evolution of the PRRS virus and the recent emergence of more pathogenic strains present continuous challenges in the development of a next generation MLV vaccines for the aspect of safety, and equal protection against homologous and heterologous virus using modern advanced biotechnologies such as PRRSV cDNA clones, replicating vector-based PRRSV vaccines, codon pairs de-optimization, chimeric PRRSVs, and DNA shuffling [10,130,131].

## Figures and Tables

**Table 1:** Major Porcine reproductive and respiratory syndrome (PRRS) modified-live virus (MLV) vaccines used in worldwide.

Species	Name	Usage	Introduction	Company
PRRSV-1	Porcilis PRRS	Sow, Piglet	2000	MSD
	UNISTRAIN PRRS	Sow, Piglet	2013	Laboratories Hipra S.A.
	ReproCyc PRRS EU	Sow	2015	Boehringer Ingelheim
	Ingelvac PRRSFLEX EU	Piglet	2015	Boehringer Ingelheim
PRRSV-2	Ingelvac PRRS MLV	Sow, Piglet	1994	Boehringer Ingelheim
	Fostera PRRS	Sow, Piglet	2012	Zoetis
	PrimePac PRRS	Sow, Piglet	2014	MSD
	Prevacent PRRS	Sow, Piglet	2018	Elanco Animal Health

**Table 2:** Efficacy of PRRS modified-live virus (MLV) vaccines against respiratory disease in growing pigs.

Type of Study	MLV Vaccines	References
Respiratory disease against PRRSV-1	Porcilis PRRS	[32,37,39,40,41,65]
	UNISTRAIN PRRS	[33,37,38]
	Ingelvac PRRSFLEX EU	[34]
	Ingelvac PRRS MLV	[34,39,65]
	Fostera PRRS	[25,49]
Respiratory disease against PRRSV-2	Porcilis PRRS	[37]
	UNISTRAIN PRRS	[37,45,47,63]
	Ingelvac PRRS MLV	[49,50,58,60]
	Fostera PRRS	[23,26,48,52,60]
	PrimePac PRRS	[62]

**Table 3:** Efficacy of PRRS MLV vaccines against reproductive failure in gilts and sows.

Type of Study	MLV Vaccines	References
Reproductive failure against PRRSV-1	Porcilis PRRS	[104,105]
	UNISTRAIN PRRS	[96,102]
	ReproCyc PRRS EU	[99]
	Ingelvac PRRS MLV	[95]
	Fostera PRRS	[103]
Reproductive failure against PRRSV-2	UNISTRAIN PRRS	[96]
	Ingelvac PRRS MLV	[95,98]
	Fostera PRRS	[94,106]
